# Serum amyloid A, protein Z, and C4b-binding protein β chain as new potential biomarkers for pulmonary tuberculosis

**DOI:** 10.1371/journal.pone.0173304

**Published:** 2017-03-09

**Authors:** Ting-Ting Jiang, Li-Ying Shi, Li-Liang Wei, Xiang Li, Su Yang, Chong Wang, Chang-Ming Liu, Zhong-Liang Chen, Hui-Hui Tu, Zhong-Jie Li, Ji-Cheng Li

**Affiliations:** 1 South China University of Technology School of Medicine, Guangzhou, P.R. China; 2 Department of Clinical Laboratory, Zhejiang Hospital, Hangzhou, P.R. China; 3 Department of Pneumology, Shaoxing Municipal Hospital, Shaoxing, P.R. China; 4 Key Laboratory of Gastroenteropathy, Zhejiang Province People’s Hospital, Hangzhou, China; 5 Institute of Cell Biology, Zhejiang University, Hangzhou, P.R. China; Food and Drug Administration, UNITED STATES

## Abstract

The aim of this study was to discover novel biomarkers for pulmonary tuberculosis (TB). Differentially expressed proteins in the serum of patients with TB were screened and identified by iTRAQ-two dimensional liquid chromatography tandem mass spectrometry analysis. A total of 79 abnormal proteins were discovered in patients with TB compared with healthy controls. Of these, significant differences were observed in 47 abnormally expressed proteins between patients with TB or pneumonia and chronic obstructive pulmonary disease (COPD). Patients with TB (n = 136) exhibited significantly higher levels of serum amyloid A (SAA), vitamin K-dependent protein Z (PROZ), and C4b-binding protein β chain (C4BPB) than those in healthy controls (n = 66) (*P*<0.0001 for each) albeit significantly lower levels compared with those in patients with pneumonia (n = 72) (*P*<0.0001 for each) or COPD (n = 72) (*P*<0.0001, *P*<0.0001, *P* = 0.0016, respectively). After 6 months of treatment, the levels of SAA and PROZ were significantly increased (*P* = 0.022, *P*<0.0001, respectively), whereas the level of C4BPB was significantly decreased (*P* = 0.0038) in treated TB cases (n = 72). Clinical analysis showed that there were significant differences in blood clotting and lipid indices in patients with TB compared with healthy controls, patients with pneumonia or COPD, and treated TB cases (*P*<0.05). Correlation analysis revealed significant correlations between PROZ and INR (rs = 0.414, *P* = 0.044), and between C4BPB and FIB (rs = 0.617, *P* = 0.0002) in patients with TB. Receiver operating characteristic curve analysis revealed that the area under the curve value of the diagnostic model combining SAA, PROZ, and C4BPB to discriminate the TB group from the healthy control, pneumonia, COPD, and cured TB groups was 0.972, 0.928, 0.957, and 0.969, respectively. Together, these results suggested that SAA, PROZ, and C4BPB may serve as new potential biomarkers for TB. Our study may thus provide experimental data for the differential diagnosis of TB.

## Introduction

Pulmonary tuberculosis (TB), caused by *Mycobacterium tuberculosis* (Mtb), poses considerable threat to human health, especially after the emergence of HIV-associated TB and multi-drug resistant TB, with an estimated 9.6 million new TB cases and 1.5 million TB deaths worldwide reported in 2014 [1]. In particular, China has the second largest number of TB cases in the world. According to the fifth national TB epidemiological survey in China, approximately 1.30 million new TB cases occur annually, accounting for 14.3% TB cases worldwide [2]. Between 2000 and 2014, an estimated 43 million lives have been saved through TB diagnosis and treatment [1]. However, delay in the diagnosis of TB may increase the severity and mortality of the disease as well as increase the risk of transmission. Therefore, early diagnosis and treatment are important to control the spread of TB [3,4].

In the clinic, the detection of TB is still heavily dependent on sputum smear, sputum culture, chest radiography (X-ray/computerized tomography (CT) scan), and clinical symptomatology [5]. However, the sputum smear positivity rate among TB cases was found to be only 20–30% [6], and numerous cases of less infectious forms of TB cannot be detected by this method. In comparison, Mtb culturing represents the gold standard for diagnosis of TB, providing a higher positive rate (30–40%) than sputum smear; however, the culture requires 4 to 8 weeks for the growth of Mtb [5]. Furthermore, the radiological findings and clinical symptoms of patients with TB in the early stage of infection are not specific and it is difficult to distinguish TB from other pulmonary diseases [5,6]. Therefore, there is an urgent need for the development of a rapid and accurate diagnostic method for the effective treatment and control of TB.

Serum protein provides important clues toward exploring the pathological and physiological conditions of the body, and the roles of serum proteins have been widely investigated in cancer and other diseases [7,8]. Notably, a series of changes in the level of serum proteins are caused by Mtb infection [9,10]. Therefore, serum proteins may serve as ideal biomarkers for the diagnosis of TB.

In the present study, differentially expressed proteins were screened and identified in patients with TB, and compared with those in patients with pneumonia or chronic obstructive pulmonary disease (COPD), and healthy controls using iTRAQ-two dimensional liquid chromatography tandem mass spectrometry (2D LC-MS/MS) and enzyme-linked immunosorbent assay (ELISA). Abnormal proteins found in patients with TB were also evaluated in the treated TB cases by ELISA. Clinical data and their correlation with the validated proteins were analyzed using statistical methods. Finally, the accuracy of the new protein biomarkers to distinguish patients with TB was analyzed using receiver operating characteristic (ROC) curves. The study will likely provide an experimental basis for biomarker research in patients with TB and may facilitate the diagnosis and prevention of this disorder.

## Material and methods

### Patients and sample collection

TB patients with the following criteria were included [9]: *a*: Positive sputum examination (smear microscopic examination or bacterial culture); *b*: Negative sputum examination, but chest X-ray and CT scan showing features of typical active TB; *c*: Pathological diagnosis of TB in lung specimens; *d*: Clinically suspected of having TB and confirmed after clinical follow-up and X-ray observations, and excluding other lung diseases; *e*: Diagnosis of tuberculous pleurisy, with other causes of pleural effusion ruled out.

**Fig 1:**
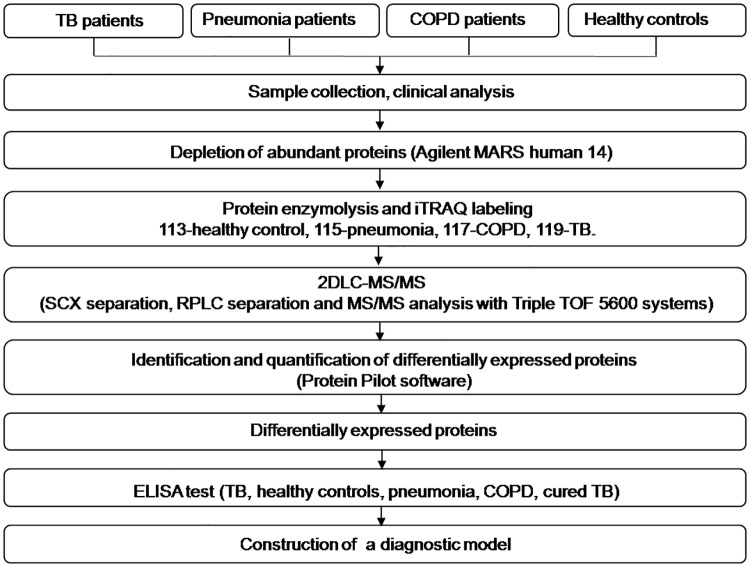
Flowchart of the experimental design. Differentially expressed proteins were identified and quantified by applying iTRAQ-2DLC-MS/MS technology and by ELISA.

The workflow of this study is illustrated in Fig 1. A total of 418 subjects were included in this study, incorporating 136 patients with pulmonary TB (78 men, 58 women; aged 18–78 years; mean age 43.80 ± 16.99 years) and 72 6-month-treated TB cases (46 men, 26 women; aged 18–78 years; mean age 41.94 ± 16.80 years) recruited from the Shaoxing Municipal Hospital (Shaoxing, Zhejiang, China). In addition, 66 healthy controls (33 men, 33 women; aged 18–77 years; mean age 41.87 ± 14.59 years) were recruited from the Zhejiang Hospital (Hangzhou, Zhejiang, China). Furthermore, 72 patients with pneumonia (39 men, 33 women; aged 18–78 years; mean age 48.71 ± 17.04 years) were recruited from the Zhejiang Provincial People’s Hospital (Hangzhou, Zhejiang, China), and 72 patients with COPD (44 men, 28 women; aged 45–80 years; mean age 68.90 ± 9.56 years) were recruited from the Zhejiang Provincial People’s Hospital and Zhejiang Provincial Tongde Hospital (Hangzhou, Zhejiang, China) (S1 Table). Patients with extra-pulmonary TB, hepatitis B, AIDS, diabetes, and other diseases as well as those using immune inhibitors were excluded from the study.

Morning fasting blood samples from subjects were collected. Serum samples were clotted in Vacutainers without anticoagulation and then centrifuged at 956 g for 10 min at 4°C to separate the serum. A portion of the serum was kept for clinical detection and the remaining was aliquoted in sterile centrifuge tubes and stored at −80°C for future use. Clinical parameters of patients with TB were assessed. The present study was approved by the Medical Ethics Committee of South China University of Technology School of Medicine (China), and written informed consent was obtained from all the blood donors prior to the commencement of this study.

### Abundant protein depletion and iTRAQ labeling

Experimental groups were as follows: healthy controls, and patients with TB, pneumonia, or COPD. To increase the accuracy and precision of the experimental data, equal amounts of 10 different serum samples from each group were mixed to produce pooled samples. We eliminated 14 high-abundancy serum proteins including albumin, IgG, antitrypsin, IgA, transferrin, haptoglobin, fibrinogen, alpha 2 macroglobulin, alpha 1 acid glycoprotein, IgM, apolipoprotein AI, apolipoprotein AII, complement C3, and transthyretin using Agilent multiple affinity removal LC column-Human 14 (MARS) (Agilent Technologies, Santa Clara, CA, USA) [8]. The protein samples were then desalted and concentrated using 3-kDa cutoff ultracentrifugation columns (Millipore, Billerica, MA, USA).

Serum samples were then reduced, digested, and labeled. iTRAQ isotope-labeling was carried out using the iTRAQ kit according to the manufacturer’s instructions (Applied Biosystems, Foster city, CA, USA). Firstly, 100 μg serum protein samples of each group were soaked with pre-chilled acetone (acetone: sample = 5:1) and precipitated for 1 h at −20°C, then centrifuged at 13201 g for 15 min. The supernatant was removed and the proteins were dissolved by 1% SDS and dissolution buffer. Secondly, the proteins were reacted with reducing agents at 60°C for 1 h and blocked with cysteine at room temperature for 10 min. The reduced protein samples were digested with trypsin (trypsin: sample = 1:20) at 37°C overnight. Finally, the digested samples were labeled using iTRAQ reagents. Specifically, 113, 115, 117, and 119 labeling reagents containing isopropanol were added into sample tubes representing the healthy control, pneumonia, COPD, and TB groups, respectively (114, 116, 118, and 121 were used to label other samples not included in this research). After reacting at room temperature for 1 h, the redundant iTRAQ reagent was decomposed by three volumes water and the labeled samples were dried using vacuum centrifugal concentration (Christ RVC 2 to 25, Osterode, Germany) [8,11].

### 2D LC-MS/MS analysis

The labeled samples were desalted with Sep-Pak Vac C18 (Waters, Milford, MA, USA), and the chromatographic separation was performed using a 20AD LC system (SHIMADZU, Kyoto, Japan). The iTRAQ- labeled peptides were then separated with a strong cation exchange LC column (2.1 mm × 100 mm, 5 microns, 200 A, Polysulfoethyl column, SCX) (Nest Group, Southborough, MA, USA). A linear gradient (0–80%) of buffer B (25% Acetonitrile, 350 mM KCL, 10 mM KH_2_PO_4_, pH 2.6) and buffer A (25% Acetonitrile, 10 mM KH_2_PO_4_, PH2.6) was applied to elute the SCX fractions. Finally, a total of 20 gradient peptides were collected based on the peak type measured by od reading at 214nm/280nm [12].

The concentrated peptides were dissolved by 20 μL RPLC phase A (5% Acetonitrile, 0.1% formic acid) (TEDIA, Fairfield, CT, USA) for 2D reversed-phase chromatographic separation. The 20 SCX fractions were then injected into the ZORBAX 300SB-C18 RP column (5 μm, 300Å, 0.1 × 150 mm, Microm, Auburn, CA, USA). The subsequent separation of SCX fractions was conducted with a linear gradient (5–80%) of phase B (95% Acetonitrile, 0.1% formic acid) and phase A. The separated fractions were analyzed using a Triple TOF 5600 system (Applied Biosystems, USA) and the mass spectra were acquired by a positive ion mode and information-dependent acquisition mode. The survey scans were obtained between 400–1500 m/z. A total of 20 most-intense multiply charged ions were chosen for MS/MS analysis with a mass range of 100–2000 m/z. Each labeled specimen was analyzed twice and expressed as run 1 and run 2.

### Protein identification and relative quantification

Proteins were directly identified and relatively quantified using ProteinPilotTM version 4.2 beta (Applied Biosystems), which applied the ProGroup algorithm to eliminate redundant hits and performed comparative quantification. The spectra data of MS/MS analysis were obtained through the International Protein Index database (version 3.87, HUMAN). To minimize false positives, the cutoff for protein identification was set as follows: unused ProtScore ≥ 1.3, and ≥ 2 peptides with 99% confidence interval (CI). The loading error and confidence level (*P* value) was calculated by Protein Pilot. The results were considered to be reliable when the error factor was < 2 and the confidence level was < 0.05 [13]. The relative quantification of differentially expressed proteins between the two groups was calculated as an average ratio. The ratios > 1.25 or < 0.8 were chosen, representing up- and down-regulated proteins, respectively.

### Bioinformatics analysis

The functional distribution of proteins including their molecular function, cellular component, and biological process was determined by an online tool based on the Gene Ontology (GO) annotation project. The protein-protein functional network was analyzed with STRING software through the net (http://string-db.org/). Pathway analysis of differentially expressed proteins was elucidated using the Kyoto Encyclopedia of Genes and Genomes database (KEGG).

### ELISA analysis

To validate the iTRAQ results, differentially expressed proteins were quantified in healthy controls, and patients with TB, pneumonia, or COPD using ELISA. To investigate the expression level of differentially expressed proteins in patients with TB after treatment, 6-month-treated TB cases were also quantified by ELISA. The human SAA ELISA kit (Abnova Co., Taipei, Taiwan) with a detection limit of 0.06 μg/mL was applied to detect SAA concentration in serum using a 1:10 dilution factor. The human PROZ ELISA kit (CUSABIO Biotech Co., Wuhan, Hubei, China) with a detection limit of 31.25 ng/mL was utilized to measure PROZ concentration in serum with a dilution factor of 1. The human C4BPB ELISA kit (CUSABIO) with a minimum detectable dose of 0.78 ng/mL was employed to detect C4BPB in serum at a 1:4000 dilution factor. ELISA assays were carried out according to the manufacturer’s instructions, in duplicate.

### Statistical analysis

All the experimental data were analyzed using SPSS software (version 16.0, Chicago, IL, USA) and GraphPad Prism 5 (GraphPad Software, Inc., La Jolla, CA USA). *P* < 0.05 was considered statistically significant. Parametric data were presented as the mean ± SD and were investigated using the chi-square test for the composition ratios and t tests for means. The non-parametric data were presented as the median ± interquartile range (IQR) and were analyzed using the Mann–Whitney U test for two groups and the Kruskal-Wallis H test for more than two groups. The Spearman correlation method was employed to explore the association between differentially expressed proteins and clinical data. The diagnostic score of patients with TB, healthy controls, patients with pneumonia or COPD, and treated TB cases was set as 0, 1, 2, 3, and 4, respectively. Biomarker Pattern Software (BPS, Ciphergen Biosystems) was used to construct the decision tree of TB with the validated proteins. ROC curves were generated by MedCalc Software (Version 12.3.0, Belgium). The area under the curve (AUC) value, sensitivity, and specificity were calculated to assess the diagnostic value of novel biomarkers.

## Results

### Identification and relative quantification of differential protein expression

**Fig 2:**
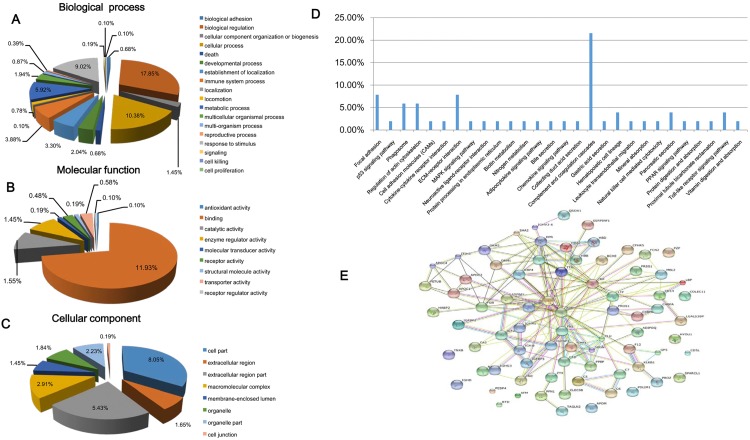
Bioinformatics analysis of differentially expressed proteins. (A) Biological process (GO analysis). (B) Molecular function (GO analysis). (C) Cellular component (GO analysis). (D) KEGG analysis. (E) Functional network of differentially expressed proteins. ECM: extracellular matrix; MAPK: Mitogen-activated protein kinase; PPAR: peroxisome proliferator activated receptor.

The fold changes of differentially expressed proteins were calculated based on the intensities of iTRAQ reporter ions. We quantified 79 abnormally expressed proteins between patients with TB and healthy controls including 51 up-regulated proteins (fold change >1.25, S2 Table) and 28 down-regulated proteins (fold change <0.8, S2 Table). The biological process (Fig 2A), molecular function (Fig 2B), and cellular component (Fig 2C) of proteins annotated by GO analysis showed broad functional distribution. It was illustrated that 17.85% of the differentially expressed proteins were related to biological regulation, 11.93% had a role in binding, 10.38% were involved in cellular processes, and 5.92% were associated with the metabolic process. According to the results of KEGG analysis, 21.57% abnormal proteins were involved in the complement and coagulation cascades, 7.84% participated in focal adhesions, and 7.84% were involved in the extracellular matrix-receptor interaction (Fig 2D). Furthermore, protein-protein functional network diagram analysis indicated that differentially expressed proteins were involved in functional connections (Fig 2E). A significant difference was observed in 47 abnormally expressed proteins between the TB group and the healthy control, pneumonia, and COPD groups (Table 1).

**Table 1:** Abnormally expressed proteins and their expression levels quantified by iTRAQ-2DLC-MS/MS analysis.

Protein ID	Abbreviation	Name	No. of peptides (>95%)	iTRAQ ratio		
				TB/ Controls	TB/ Pneumonia	TB/ COPD
P00738	HPT	Haptoglobin	254	62.44	0.54	0.38
P68871	HBB	Hemoglobin subunit beta	50	57.22	0.65	0.42
P04003	C4BPA	C4b-binding protein alpha chain	32	20.99	1.90	1.70
P0CG05	LAC2	Ig lambda-2 chain C regions	10	20.44	0.21	0.44
P01871	IGHM	Ig mu chain C region	17	16.44	0.51	0.21
P01860	IGHG3	Ig gamma-3 chain C region	19	14.67	0.10	0.27
P69905	HBA	Hemoglobin subunit alpha	14	13.59	0.36	0.41
P01876	IGHA1	Ig alpha-1 chain C region	10	11.77	0.05	1.31
P02735	SAA	Serum amyloid A protein	30	9.34	0.13	0.56
P20851	C4BPB	C4b-binding protein beta chain	17	8.81	0.70	0.25
P02675	FIBB	Fibrinogen beta chain	12	8.39	1.29	0.33
P02763	A1AG1	Alpha-1-acid glycoprotein 1	50	7.76	4.47	1.80
P19652	A1AG2	Alpha-1-acid glycoprotein 2	26	6.96	5.00	1.47
P01834	IGKC	Ig kappa chain C region	10	6.59	0.07	0.32
P02042	HBD	Hemoglobin subunit delta	36	5.86	0.60	0.50
P20742	PZP	Pregnancy zone protein	77	5.73	9.90	2.43
P07225	PROS	Vitamin K-dependent protein S	36	3.73	2.13	2.00
P02766	TTHY	Transthyretin	5	3.58	0.12	0.34
Q14520	HABP2	Hyaluronan-binding protein 2	10	3.54	9.96	1.65
P08571	CD14	Monocyte differentiation antigen CD14	21	2.62	2.43	3.94
B9A064	IGLL5	Immunoglobulin lambda-like polypeptide 5	7	2.49	0.56	0.23
Q15848	ADIPO	Adiponectin	11	2.39	0.56	0.55
Q9UGM5	FETUB	Fetuin-B	20	2.30	2.73	3.48
P11226	MBL2	Mannose-binding protein C	18	2.25	4.92	1.65
P37802	TAGL2	Transgelin-2	11	2.01	5.64	4.14
P24821	TENA	Tenascin	12	1.95	0.65	0.77
P02751	FINC	Fibronectin	64	1.85	4.17	1.83
P18428	LBP	Lipopolysaccharide-binding protein	20	1.74	17.31	0.58
P00918	CAH2	Carbonic anhydrase 2	4	1.68	1.77	0.67
Q08380	LG3BP	Galectin-3-binding protein	9	1.64	0.53	0.75
P01344	IGF2	Insulin-like growth factor II	3	1.63	2.08	1.56
O00151	PDLI1	PDZ and LIM domain protein 1	7	1.63	1.68	1.77
P10124	SRGN	Serglycin	11	1.62	1.99	1.68
P07737	PROF1	Profilin-1	5	1.62	4.11	2.00
Q9Y4L1	HYOU1	Hypoxia up-regulated protein 1	5	1.54	6.05	0.78
P22891	PROZ	Vitamin K-dependent protein Z	2	1.31	0.79	0.44
P03952	KLKB1	Plasma kallikrein	54	0.60	4.23	0.66
P02654	APOC1	Apolipoprotein C-I	19	0.57	2.92	0.46
P13671	CO6	Complement component C6	83	0.54	0.74	0.26
O95445	APOM	Apolipoprotein M	9	0.50	0.19	0.33
P02753	RET4	Retinol-binding protein 4	79	0.48	2.78	0.42
P43652	AFAM	Afamin	65	0.47	12.89	0.76
P22105	TENX	Tenascin-X	25	0.43	0.65	0.72
P02775	CXCL7	Platelet basic protein	26	0.43	0.41	0.51
P02788	TRFL	Lactotransferrin	10	0.20	5.29	1.47
P02655	APOC2	Apolipoprotein C-II	47	0.18	0.68	0.14
P02768	ALBU	Serum albumin	27	0.02	0.02	2.00

TB: pulmonary tuberculosis; H: healthy controls. Peptides (>95%): the number of peptides with a coverage of 95%.

After synthesizing the above results including fold change ratio and GO, KEGG, and STRING bioinformatics analyses, we observed that the most significantly expressed proteins were associated with the complement and coagulation cascades pathway. We therefore focused on the proteins involved in this pathway, among which C4BPB, vitamin K-dependent protein S (PROS), C4BPA, and FIBB were the most significantly expressed. Although SAA and PROZ are not involved in this pathway, they are closely associated with blood coagulation. As the serum level of PROS has been investigated previously by Kager et al. [14] in patients with TB and commercial ELISA kits for C4BPA and FIBB were unavailable, we selected SAA, PROZ, and C4BPB for further study. SAA is an acute phase protein that is linked to the thrombotic complications of TB through the regulation of tissue factor. PROZ, a vitamin K-dependent glycoprotein, is associated with the prevention of thrombin generation in the early stage of coagulation. C4BPB can specifically bind to PROS and participate in the inactivation of coagulation factors Va and VIIIa, and inhibit thrombin generation. The MS/MS spectra of SAA, PROZ, and C4BPB are shown in the supporting information (S1 Fig).

### ELISA verification of differentially expressed proteins

**Fig 3:**
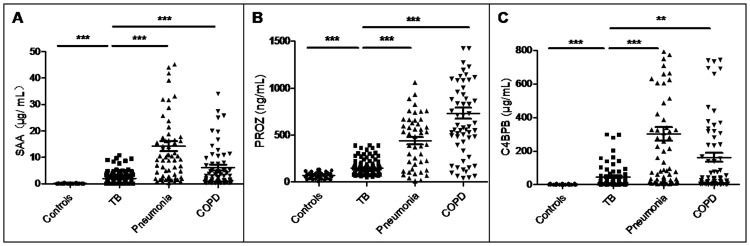
Serum proteins levels among the patients with TB, healthy controls, and patients with pneumonia or COPD. (A) SAA. (B) PROZ. (C) C4BPB. TB: pulmonary tuberculosis. *P* value <0.05 indicates statistical significance using the Mann-Whitney U test. *P < 0.05, **P < 0.01, ***P < 0.001.

**Fig 4:**
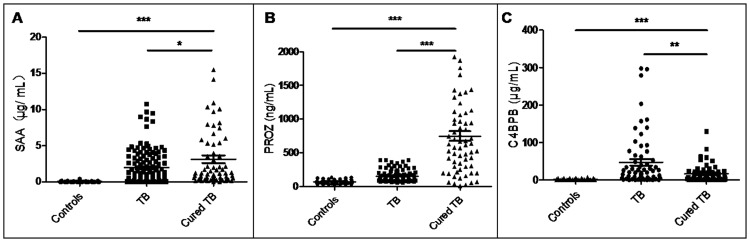
Serum proteins levels in the healthy controls, patients with TB, and treated TB cases. (A) SAA. (B) PROZ. (C) C4BPB. TB: pulmonary tuberculosis. *P* value <0.05 indicates statistical significance using the Mann-Whitney U test. *P < 0.05, **P < 0.01, ***P < 0.001.

The three new candidate biomarkers SAA, PROZ, and C4BPB were validated by ELISA in 136 patients with TB, 66 healthy controls, 72 patients with pneumonia, and 72 patients with COPD. The serum concentrations of SAA, PROZ, and C4BPB in patients with TB were significantly higher than those in healthy controls (*P* < 0.0001, *P* < 0.0001, *P* < 0.0001, Fig 3) but were significantly lower than those in patients with pneumonia (*P* < 0.0001 for each, Fig 3) or COPD (*P* < 0.0001, *P* < 0.0001, *P* = 0.0016, respectively, Fig 3). Furthermore, we also quantified the serum concentrations of these proteins in 72 treated TB cases. The serum levels of SAA and PROZ were significantly increased in treated TB cases compared with those in patients with TB (*P* = 0.022, *P* < 0.0001, respectively, Fig 4) whereas the serum level of C4BPB was significantly decreased (*P* = 0.0038, Fig 4). However, the C4BPB serum level was significantly higher in treated TB cases than in controls (*P* < 0.0001, Fig 4).

According to their clinical characteristics, all the patients with TB were separated based on age, gender, sputum smear test, lung lesion, and chest CT scan/X-ray results. Significantly increased SAA and PROZ levels were observed in patients with TB having cavitary lung lesions (*P* = 0.006, *P* = 0.03, respectively, Table 2), compared with those having non-cavitary lung lesions. In addition, the serum concentrations of SAA were higher in patients with TB and double lung lesions than in those with single lung lesions (*P* = 0.003, Table 2). The serum SAA levels were also found to be increased in smear positive than smear negative patients with TB (*P* = 0.02, Table 2).

**Table 2:** Average serum levels of proteins according to the clinical characteristics of patients with TB.

Clinical characteristics (Cases)	SAA (μg/mL)	*P* value	PROZ (ng/mL)	*P* value	C4BPB (μg/mL)	*P* value
**Age**		0.15		0.36		0.52
18–34 (51)	2.54 ± 3.69		190.12 ± 83.50		45.33 ± 50.62	
35–49 (30)	1.94 ± 2.79		238.57 ± 114.98		33.73 ± 22.53	
≥50 (55)	1.56 ± 2.79		198.12 ± 160.35		44.00 ± 39.16	
**Gender**						
Male (78)	1.88 ± 2.94	0.48	157.44 ± 78.97	0.06	50.79 ± 72.36	0.34
Female (58)	2.09 ± 3.13		262.90 ± 211.70		29.33 ± 24.28	
**Sputum smear**						
Positive (114)	2.21 ± 3.46	0.02*	200.49 ± 129.97	0.52	41.17 ± 42.70	0.98
negative (22)	0.93 ± 1.36		307.446 ± 318.35		59.83 ± 26.47	
**Lung lesion**						
Double (78)	2.41 ± 3.58	0.003**	237.87 ± 161.86	0.14	47.94 ± 49.04	0.43
Single (58)	1.28 ± 1.34		157.22 ± 91.90		38.75 ± 30.89	
**Chest CT/X-ray**						
Cavitary (57)	2.72 ± 3.87	0.006**	224.79 ± 133.63	0.03*	42.99 ± 54.59	0.6
Non-cavitary (79)	1.50 ± 2.51		201.52 ± 75.33		42.00 ± 34.69	

All data are presented as the median ± IQR. The *P* value of two groups was analyzed using the Mann-Whitney U test, and that among three groups was tested using the Kruskal-Wallis H test.

*P < 0.05,

**P < 0.01.

### Clinical analysis

Significant differences between patients with TB and healthy controls were observed in the following clinical indices (*P* < 0.05, Table 3): plasma prothrombin time (PT), international normalized ratio (INR), plasma fibrinogen (FIB), activated partial thromboplastin time (APTT), thromboplastin time (TT), total cholesterol, high density lipoprotein cholesterol (HDL-C), and low density lipoprotein cholesterol (LDL-C). There were significant differences in total protein, globulin, FIB, APTT, TT, HDL-C, apolipoprotein A1 (APOA1), apolipoprotein B (APOB), and lipoprotein between patients with TB and those with pneumonia (*P* < 0.05, Table 3). Similarly, significant differences in total protein, albumin, FIB, APTT, TT, total cholesterol, HDL-C, APOA1, and APOB were observed between patients with TB and those with COPD (*P* < 0.05, Table 3). There were significant differences in albumin, albumin/globulin (A/G), HDL-C, and APOA1 between patients with TB and treated TB cases (*P* < 0.05, Table 3).

**Table 3:** Clinical data of patients with TB, healthy controls, patients with pneumonia or COPD, and treated TB cases.

	TB (N = 136)	Control (N = 66)	Pneumonia (N = 72)	COPD (N = 72)	Treated TB (N = 72)	*P* value			
						TB vs Control	TB vs Pneumonia	TB vs COPD	TB vs Treated TB
Total protein (g/L)	71.24 ± 6.07	ND	69.12 ± 6.67	66.34 ± 6.33	73.76 ± 5.64	**/**	0.042*	< 0.0001***	0.053
Albumin (g/L)	41.28 ± 4.89	ND	44.83 ± 41.03	37.78 ± 4.24	43.49 ± 4.14	**/**	0.485	< 0.0001***	0.021*
Globulin (g/L)	29.96 ± 4.87	ND	28.42 ± 4.32	28.56 ± 5.00	28.99 ± 4.53	**/**	0.037*	0.086	0.320
Albumin/globulin	1.41 ± 0.28	ND	1.46 ± 0.28	1.36 ± 0.27	1.54 ± 0.27	**/**	0.338	0.255	0.036*
PT (s)	13.64 ± 1.48	10.18 ± 0.60	14.05 ± 2.22	14.07 ± 2.85	ND	< 0.0001***	0.120	0.178	**/**
INR	1.06 ± 0.16	1.01 ± 0.06	1.11 ± 0.19	1.10 ± 0.20	ND	0.0189*	0.246	0.329	**/**
FIB (g/L)	4.25 ± 1.73	3.49 ± 0.61	5.54 ± 2.17	5.52 ± 2.71	ND	0.0008***	< 0.0001***	0.0001***	**/**
APTT (s)	29.60 ± 3.58	31.87 ± 2.34	32.23 ± 4.86	30.84 ± 4.87	ND	< 0.0001***	< 0.0001***	0.047*	**/**
TT (s)	14.92 ± 1.63	22.63 ± 1.48	15.80 ± 1.70	16.15 ± 1.83	ND	< 0.0001***	0.0004***	< 0.0001***	**/**
Total cholesterol (mmol/L)	3.67 ± 0.97	4.64 ± 0.53	3.85 ± 0.92	4.15 ± 1.05	4.12 ± 1.11	< 0.0001***	0.204	0.002**	0.131
Triglyceride (mmol/L)	1.03 ± 0.46	0.94 ± 0.32	1.06 ± 0.49	0.96 ± 0.80	0.84 ± 0.35	0.051	0.713	0.405	0.144
HDL-C (mmol/L)	1.03 ± 0.36	1.44 ± 0.26	1.18 ± 0.39	1.34 ± 0.43	1.44 ± 0.48	< 0.0001***	0.006**	< 0.0001***	0.0003***
LDL-C (mmol/L)	2.29 ± 0.93	2.73 ± 0.52	2.27 ± 0.71	2.42 ± 0.71	2.39 ± 0.80	< 0.0001***	0.904	0.289	0.702
APOA1 (g/L)	1.03 ± 0.23	ND	1.14 ± 0.31	1.19 ± 0.29	1.26 ± 0.39	**/**	0.0066**	< 0.0001***	0.003**
APOB (g/L)	0.75 ± 0.21	ND	0.85 ± 0.21	0.90 ± 0.24	0.86 ± 0.25	**/**	0.0011**	< 0.0001***	0.095
Lipoprotein (a) (mg/L)	237.83 ± 238.28	ND	155.75 ± 148.16	194.06 ± 156.01	226.42 ± 180.27	**/**	0.0052**	0.147	0.868
CRP (mg/L)	28.81 ± 38.98	ND	28.98 ± 43.56	33.55±46.74	28.52±32.51	**/**	0.978	0.464	0.980
Complement 4 (mg/L)	320.67 ± 90.22	ND	307.82 ± 103.77	293.95 ± 89.14	348.25 ± 132.70	**/**	0.679	0.251	0.572

All data are presented as the means ± SD. A/G: albumin/globulin ratio; PT: Plasma prothrombin time; INR: International normalized ratio; FIB: Plasma fibrinogen; APTT: Activated partial thromboplastin time; TT: Thromboplastin time; HDL-C: high-density lipoprotein cholesterol; LDL-C: low-density lipoprotein cholesterol; APOA1: apolipoprotein A1; APOB: apolipoprotein B; CRP: C reactive protein; ND: not detected. The *P* value between two groups was analyzed using the t-test.

*P < 0.05,

**P < 0.01,

***P < 0.001.

Furthermore, according to the Spearman correlation analysis between differentially expressed proteins and clinical indices, PROZ showed significant positive correlation with INR (rs = 0.414, *P* = 0.044, Table 4). In addition, a significant positive correlation was observed between C4BPB and fibrinogen (rs = 0.617, *P* = 0.0002, Table 4).

**Table 4:** Correlation analysis of differentially expressed proteins and clinical coagulation indices.

		**SAA**	**PROZ**	**C4BPB**	**PT**	**INR**	**APTT**	**TT**	**FIB**
SAA	r_s_	1	0.195	.	0.040	0.118	−0.024	−0.083	−0.076
	*P*_a_	.	0.061	.	0.706	0.600	0.816	0.436	0.472
PROZ	r_s_		1	.	0.133	0.414*	0.168	0.088	0.083
	*P*_a_		.	.	0.182	0.044	0.092	0.384	0.411
C4BPB	r_s_			1	0.281	0.346	0.064	−0.331	0.617**
	*P*_a_			.	0.126	0.090	0.734	0.074	0.0002
PT	r_s_				1	0.997**	0.085	0.254**	0.195*
	*P*_a_				.	0	0.312	0.003	0.020
INR	r_s_					1	0.459**	0.057	0.007
	*P*_a_					.	0.001	0.686	0.960
APTT	r_s_						1	−0.004	0.016
	*P*_a_						.	0.966	0.850
TT	r_s_							1	−0.106
	*P*_a_							.	0.218
FIB	r_s_								1
	*P*_a_								.

*P < 0.05.

**P < 0.01.

The range of rs from −0.3 to −0.1 or 0.1 to 0.3 indicates weak correlation; the range of rs from −0.5 to −0.3 or 0.3 to 0.5 indicates moderate correlation; and the range of rs from −0.7 to −0. 5 or 0.5 to 0.7 indicates significant correlation.

### ROC analysis and decision tree establishment

**Fig 5:**
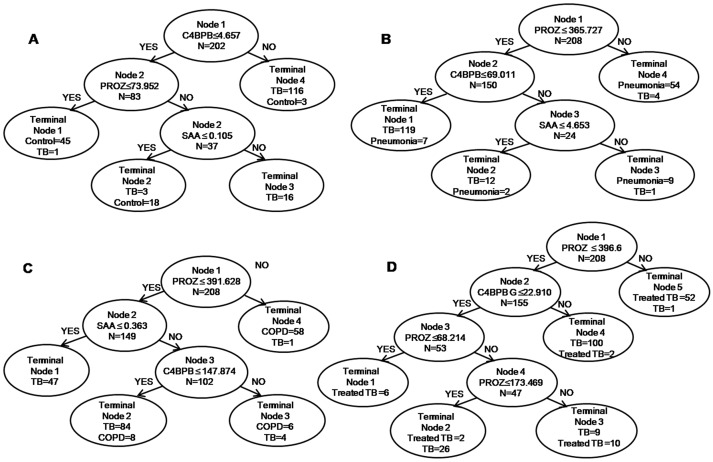
Decision trees in the diagnostic model for TB generated by Biomarker Patterns Software. Serum proteins SAA, PROZ, and C4BPB were incorporated to establish a decision tree using Biomarker Patterns Software. The diagnostic model shows the sample distribution and tree structure of the set. (A) Diagnostic model for patients with TB and healthy controls. (B) Diagnostic model for patients with TB or pneumonia. (C) Diagnostic model for patients with TB or COPD. (D) Diagnostic model for patients with TB and treated TB cases.

ROC analysis was next used to evaluate the proteins. When the individual protein served as a candidate biomarker, the AUCs of SAA, PROZ, and C4BPB were 0.843, 0.852, and 0.860, respectively, to discriminate between patients with TB and healthy controls; 0.852, 0.796, and 0.787 to discriminate between patients with TB and those with pneumonia, or 0.693, 0.884, and 0.662 for those with COPD; and 0.601, 0.867, and 0.654, respectively, to discriminate between patients with active TB and treated TB cases. However, the decision tree established through combining SAA, PROZ, and C4BPB as a panel was more accurate than those through the individual serum proteins (Table 5). The AUC was 0.972 with 97.06% sensitivity and 95.45% specificity to discriminate between patients with TB and healthy controls; 0.928 with 96.32% sensitivity and 87.50% specificity to discriminate patients with TB from those with pneumonia; or 0.957 with 96.32% sensitivity and 88.89% specificity for those with COPD patients; and 0.969 with 92.65% sensitivity and 94.44% specificity to discriminate between patients with TB and treated TB cases (Fig 5).

**Table 5:** Receiver operating characteristics of SAA, PROZ, and C4BPB in distinguishing patients with TB from healthy controls, patients with pneumonia or COPD, and treated TB cases individually and as a panel.

Protein	Sensitivity	Specificity	AUC	*P* value
*Distinguishing patients with TB from healthy controls*				
SAA	69.70%	96.40%	0.843	<0.0001
PROZ	90.40%	64.80%	0.852	<0.0001
C4BPB	69.20%	98.30%	0.860	<0.0001
C4BPB+PROZ+SAA	97.06%	95.45%	0.972	<0.0001
*Distinguishing patients with TB or pneumonia*				
SAA	91.60%	64.30%	0.852	<0.0001
PROZ	97.10%	60.90%	0.796	<0.0001
C4BPB	95.40%	48.50%	0.787	<0.0001
C4BPB+PROZ+SAA	96.32%	87.50%	0.928	<0.0001
*Distinguishing patients with TB or COPD*				
SAA	35.60%	98.60%	0.693	<0.0001
PROZ	100%	77.40%	0.884	<0.0001
C4BPB	90.80%	38.10%	0.662	0.0008
C4BPB+PROZ+SAA	96.32%	88.89%	0.957	<0.0001
*Distinguishing patients with TB from treated TB cases*				
SAA	30.30%	97%	0.601	0.0165
PROZ	100%	69.20%	0.867	<0.0001
C4BPB	46.20%	79.60%	0.654	0.0019
C4BPB+PROZ+SAA	92.65%	94.44%	0.969	<0.0001

AUC: area under the curve.

## Discussion

Common symptoms of active TB such as chest pain, weakness, cough with sputum and occasional blood, weight loss, fever, and night sweats may also occur with pneumonia and COPD. Therefore, it is difficult to differentiate patients with TB from those with pneumonia or COPD, especially when the results of sputum smear and Mtb culture, the primary current means of TB diagnostics, are negative. Furthermore, the detection rate of sputum smear is low and Mtb culture is time-consuming [5,6]. Although nucleic acid amplification techniques have been developed in recent years to diagnose TB, false positive results have been discovered in patients with bronchogenic carcinoma and individuals with a history of TB when using this technique [5]. Immunological methods such as tuberculin skin test and interferon-γ release assays have also been used in the clinic to diagnose TB but these methods cannot differentiate active TB from latent TB infection [15,16].

To address these deficiencies, serum protein biomarkers for TB diagnosis have recently been studied. In particular, Agranoff et al. [17] identified two peptides (SAA protein and transthyretin) using weak cation exchange protein chip arrays coupled with surface-enhanced laser desorption ionization time of flight MS (SELDI-TOF MS) technology. This allowed the establishment of a diagnostic model combining SAA, transthyretin, CRP and neopterin that showed good accuracy. However, although the proteomic technique used was advanced and the findings were novel at that time, many serum proteins cannot be captured by weak cation exchange protein chip arrays. In addition, the reproducibility of peak intensity was poor and protein identification cannot be made based solely on SELDI-TOF MS spectra. Furthermore, serum protein biomarkers were not detected in patients with treated TB or those with other lung diseases [17,18].

In comparison, we previously discovered four specific protein peaks (2554.6, 4824.4, 5325.7, and 8606.8 Da) associated with active TB in our laboratory and established a classification model to distinguish patients with TB from healthy controls [10]. We also found that protein S100-A9, extracellular superoxide dismutase [Cu-Zn], and matrix metalloproteinase 9 may represent potential serological markers for TB to distinguish patients with this disorder from healthy controls or those with pneumonia or lung cancer patients [19]. However, the serum protein levels were not ascertained in differential diagnosis groups such as patients with COPD or treated TB cases in either study, and the latter analysis was conducted on only a small number of patients.

In the current study, we identified 47 differentially expressed proteins in patients with TB compared with healthy controls or patients with pneumonia or COPD using iTRAQ-2DLC-MS/MS. Identified serum proteins in patients with TB were found to be involved in biological regulation, binding, and metabolic processes. In addition, a large amount of differentially expressed proteins participated in the complement and coagulation cascades. C4BPB, PROS, C4BPA, and FIBB were found to be the most significantly altered proteins among this category. Additionally, although differentially expressed proteins such as SAA and PROZ were not involved in the complement and coagulation cascades, these are closely associated with coagulation [20–22]. Statistical analysis of clinical data showed that there were significant differences in blood coagulation and lipid indices in patients with TB compared with healthy controls, patients with pneumonia or COPD, and treated TB cases (*P* < 0.05). For example, significant differences in PT, INR, APTT, FIB, TT, total cholesterol, HDL-C and LDL-C were observed between patients with TB and healthy controls, suggesting that the blood coagulation and lipid indices associated with TB were abnormal. The increased serum concentration of PROS (Table 1), which can inhibit thrombin generation and stimulate fibrinolysis [23], indicated that patients with TB were in a systemic hypercoagulable state [14]. These findings were consistent with other studies [14]. The serum concentrations of C4BPA and FIBB were significantly up-regulated in patients with TB compared to controls based on the results of iTRAQ-2DLC-MS/MS (Table 1), although commercial ELISA kits were unavailable to verify these findings. C4BPB, SAA, and PROZ were therefore chosen for further study. ELISA analysis revealed that serum C4BPB, SAA, and PROZ in patients with TB differed significantly from healthy controls, patients with pneumonia or COPD, and treated TB cases (Figs 3 and 4).

The fibrinolytic system has been shown to be up regulated in chronic Mtb infection [24]. Hemoptysis occurs upon pulmonary circulation or bronchial erosion, or following pseudoaneurysm formation, leading to hemodynamic collapse in patients with TB [25]. SAA, a major acute phase protein, has been shown to enhance the expression and activity of tissue factor (TF) and inhibit the expression and secretion of TF pathway inhibitor (TFPI) [20]. In turn, TF can bind with clotting factor VII and activate factor X, leading to the formation of thrombin and promotion of the clotting cascade, which is closely linked to the thrombotic complications of TB [20,26,27]. The inhibition of TFPI concurrently diminishes its binding with the TF/VII A complex and consequent inhibition of the clotting process [20,28]. Consistent with this, it has been demonstrated that increased SAA is associated with hypercoagulability [29] and can predict cardiovascular risk in humans [20,30,31]. It has also been reported that patients with TB are more likely to experience intrinsic coagulation than healthy individuals [14,24,32]. Furthermore, thrombotic complications such as disseminated intravascular coagulation (DIC) and deep vein thrombosis can occur in TB [14,24,32]. In the present study, the serum level of SAA was significantly increased in patients with TB compared to healthy controls (*P* < 0.0001, Fig 3). Therefore, we speculated that increased SAA may be associated with the hypercoagulable state and clotting tendency in response to the up-regulated fibrinolytic system and hemoptysis in these patients.

We further found that SAA was significantly elevated in smear positive compared to smear negative TB (*P* = 0.02, Table 2). We suspected that smear positive patients were infected with high numbers of Mtb [33], which might induce greater stimulus on the fibrinolytic system and lead to increased clotting tendency and higher level of SAA. SAA was also significantly increased in patients with TB exhibiting cavitary lung lesions (*P* = 0.006, Table 2) compared to those with non-cavitary lung lesions, and a significantly increased level of SAA was observed in patients with TB presenting with double rather than single lung lesions (*P* = 0.003, Table 2). Accordingly, we suggested that more erosion occurred in TB concomitant with cavitary and double lung lesions than in patients with non-cavitary and single lung lesions [25], leading to higher levels of SAA.

PROZ, also known as protein Z, is a vitamin K-dependent glycoprotein. It exists in the form of a PROZ/ZPI complex with PROZ-dependent protease inhibitor (ZPI), and the PROZ/ZPI complex can inhibit the activation of factor X a in the presence of SERPINA10, Ca^2+^, and phospholipids [34,35], and prevent thrombin generation in the early stage of coagulation [36,37]. High levels of PROZ have been found in patients with ischemic stroke [21,22]. Increased level of protein Z has also been discovered in colon cancer and may contribute to the anticoagulant function [38]. Notably, TB is associated with a systemic hypercoagulable state [14]. In the current study, the concentration of PROZ was found to be significantly higher in patients with TB than controls (*P* < 0.0001, Fig 3). Furthermore, PROZ levels were significantly correlated to INR levels (rs = 0.414, *P* = 0.044, Table 4), which were within normal range in patients with TB but were higher than those of controls (*P* = 0.0189, Table 3). As increased INR is associated with deep venous thrombosis and pulmonary embolus formation [39], these results suggested that an elevated PROZ level may be associated with the regulation of clotting function in patients with TB. Specifically, increased PROZ may inhibit thrombin generation and prevent coagulation to mitigate the risk of thrombotic complications in these patients. In addition, increased PROZ was also found significantly increased in patients with TB including cavitary lung lesions (*P* = 0.03, Table 2), whom we assumed would have a higher risk for hemoptysis [25], than in those with non-cavitary lung lesions. The higher risk for hemoptysis might lead to increased clotting tendency and higher level of PROZ.

C4BPB can specifically bind to PROS and form a C4BP-PROS complex to participate in the inactivation of coagulation factors Va and VIIIa [23,40]. Specifically, free PROS (fPS) is a cofactor of activated protein C (APC) [41,42]. The combination of fPS and APC can cause the inactivation of factors Va and VIIIa and inhibit thrombin generation [41,42]. In turn, the concentration of C4BPB can affect the level of fPS and has been shown to serve as a marker for fPS levels [23,41,43]. Notably, the plasma concentration of protein C4BPB has been found to be increased in patients with sepsis and other patients with an acute phase response [44,45]. In addition, Sa´nchez-Pernaute et al. [41] found that C4BPB was over-expressed in the synovial membranes of patients with rheumatoid arthritis and indicated that C4BPB was involved in the inactivation of the protein C anti-coagulatory pathway by coupling with protein S, thus contributing to the restoration of tissue integrity.

In the current study, the concentration of C4BPB in patients with TB was significantly higher than that in healthy controls (*P* < 0.0001, Fig 3). Furthermore, C4BPB levels were significantly correlated to those of FIB (rs = 0.617, *P* = 0.0002, Table 4), which were within normal range in patients with TB but were higher than those in healthy controls (*P* = 0.0008, Table 3). In particular, cytokine-mediated induction of the over-expression of the coagulation protein FIB is indicative of the coagulation tendency in patients with TB [10,46,47]. Therefore, we speculated that the elevated C4BPB in these patients may be associated with the regulation of their coagulation tendency, as the elevated C4BPB reflects the increased level of fPS [23,41,43], which can affect anti-clotting function by inhibiting the production of thrombin [41,42]. In addition, C4BPB can also regulate complement activity [48]. For example, an increase in serum complement C4b, which is produced following activation of the mannose binding lectin pathway (a part of the complement system), has been observed in TB along with the induction of tissue damage [9]. Conversely, increased C4BPB can hydrolyze the complement C4b and reduce tissue damage [49].

Notably, the pulmonary disorders pneumonia and COPD are also linked to an increased risk of vascular diseases including artery and venous thrombosis [50,51]. Furthermore, Lannergård et al. [52] found that patients with a short duration of community-acquired pneumonia prior to hospital admission exhibited increased levels of SAA and Bozinovski et al. [53] found that SAA was markedly up-regulated in patients with COPD and that this upregulation was associated with glucocorticosteroid-resistant lung inflammation in these patients. However, few studies are available regarding the serum concentration of PROZ and C4BPB in patients with pneumonia or COPD. In the present study, we found that the serum levels of SAA, PROZ, and C4BPB were significantly decreased in patients with TB compared to those with pneumonia or COPD (Fig 3), indicating that a larger alteration of proteins related to the coagulation system occurred in patients with pneumonia or COPD than in those with TB.

In comparison, SAA and PROZ levels were significantly higher in treated TB cases than in patients with TB (Fig 4) whereas C4BPB was significantly decreased in treated TB cases (*P* = 0.0038, Fig 4) although it remained significantly higher than that in controls (Fig 4). These results indicated that protein related abnormalities in the blood coagulation system of patients with TB were still present after 6 months of conventional treatment. Bronchiectasis is often found after the treatment of TB in the clinic and patients with bronchiectasis often experience hemoptysis [54]. Therefore, the abnormalities in the blood coagulation system of treated TB cases may be caused by bronchiectasis. Alternatively, it should be noted that the TB treatment consisted of isoniazid, rifampin, ethambutol, and pyrazinamide for the first two months followed by isoniazid and rifampin for another four months; non-TB drugs were not used during the six-month course of treatment. Blood samples of patients cured of TB were collected after six-month treatment without any drug withdrawal time. It has been reported that rifampin therapy could cause DIC and may even deteriorate the clinical course of DIC in patients with TB [55,56]. Therefore, the serum levels of SAA in patients cured of TB may be associated with the hypercoagulable state and clotting tendency caused by rifampin-induced DIC whereas the increased level of PROZ may have a role in preventing the formation of this complication.

Finally, although individual biomarkers might have insufficient diagnostic accuracy because of the biological complexity of TB, a diagnostic model in combination with the use of several biomarkers offers the possibility of enhanced sensitivity and specificity. For example, a decision tree represents an integrative method for discriminating clinical course and/or pathological features that has been applied in biomarker studies of lymphoma, acute leukemia, and multidrug-resistant (MDR)-TB [57,58]. In particular, Wang et al. [58] identified six potential biomarkers for MDR-TB and established decision trees to improve the sensitivity and specificity in discriminating between MDR-TB and TB. In the present study, we established decision tree diagnostic models consisting of SAA, PROZ, and C4BPB to discriminate patients with TB from healthy controls, patients with pneumonia or COPD, and treated TB cases (Fig 5). The sensitivity and specificity of the diagnostic model to discriminate patients with TB from healthy controls were 97.06% and 95.45%, respectively; from patients with pneumonia were 96.32% and 87.50%; from patients with COPD were 96.32% and 88.89%; and from treated TB cases were 92.65% and 94.44%, respectively (Table 5). Therefore, SAA, PROZ, and C4BPB may be suitable as new potential diagnostic biomarkers for pulmonary TB.

## Conclusions

In this study, three potential diagnostic biomarkers (SAA, PROZ, and C4BPB) for TB were acquired by iTRAQ-2DLC-MS/MS and ELISA. When analyzed in combination, these biomarkers yielded better specificity and sensitivity than obtained following individual assessment to discriminate patients with TB from healthy controls, patients with pneumonia or COPD, and treated TB cases. Furthermore, the abnormal levels of SAA, PROZ, and C4BPB were associated with the aberrant change and regulation of clotting function in patients with TB. The results of the present study may provide the underlying experimental data necessary to establish a method for the differential diagnosis of TB.

## Supporting information

S1 Table Demographic characteristics of patients with TB, healthy controls, patients with pneumonia or COPD, and treated TB cases. (DOCX)

S2 Table Abnormally expressed proteins and their expression levels between patients with TB and healthy controls quantified by iTRAQ-2DLC-MS/MS. (DOCX)

S1 Fig MS/MS spectra for the identification and quantitation of SAA, PROZ, and C4BPB. (A) The peptide sequence FFGHGAEDSLADQAANEWGR for SAA identification. (B) The sequence APDLQDLPWQVK for the identification of PROZ. (C) The peptide sequence SDAEHCPELPPVDNSIFVAK for C4BPB identification. The ion assignments were as follows: 113, healthy controls; 115, pneumonia group; 117, COPD group; 119, TB group. Note: 114, 116, 118, and 121 were used for other diseases not included in this study.
(TIF)
