# Supplementary material for: Serum amyloid A, protein Z, and C4b-binding protein β chain as new potential biomarkers for pulmonary tuberculosis
Source: PLoS One. 2017 Mar 9;12(3):e0173304. doi: 10.1371/journal.pone.0173304 (PMC5344400; doi:10.1371/journal.pone.0173304)
Supplement: S1 Table — (DOCX) [file pone.0173304.s001.docx]

**Supporting Information**

**S1 Table. Demographic characteristics of patients with TB, healthy controls, patients with pneumonia or COPD, and treated TB cases.**

|  | **TB** | **Controls** | **Pneumonia** | **COPD** | **Cured TB** |
| --- | --- | --- | --- | --- | --- |
|  | **(N=136)** | **(N=66)** | **(N=72)** | **(N=72)** | **(N=72)** |
| Age, Age range (Mean ± SD) | 18-78  (43.80±16.99) | 18-77  (41.87±14.59) | 18–78  (48.71±17.04) | 45–80  (68.90±9.56) | 18–78  (41.94±16.80) |
| Gender (female: male) | 58/78 | 33/33 | 33/39 | 28/44 | 26/46 |
| Positive sputum, no. (%) | 114(83.82) | ND | ND | ND | ND |
| Tuberculin skin test (>10 mm), no. (%) | 85(62.50) | ND | ND | ND | ND |
| Current Smoker, no. (%) | 52(38.24) | 24(36.36) | 29(40.28) | 31(43.06) | 28(38.89) |
| BCG vaccination, no. (%) | 122(89.71) | 62(93.94) | 67(93.06) | 65(90.28) | 62(86.11) |
| HIV-negative, no. (%) | 136(100) | 66(100) | 72(100) | 72(100) | 72(100) |

TB: tuberculosis; N: number of subjects; ND: not determined; BCG, Bacille Calmette-Guerin vaccine.
