# Supplementary material for: Serum amyloid A, protein Z, and C4b-binding protein β chain as new potential biomarkers for pulmonary tuberculosis
Source: PLoS One. 2017 Mar 9;12(3):e0173304. doi: 10.1371/journal.pone.0173304 (PMC5344400; doi:10.1371/journal.pone.0173304)
Supplement: S2 Table — (DOCX) [file pone.0173304.s002.docx]

**Supporting Information**

**S2 Table. Abnormally expressed proteins and their expression levels between patients with TB and healthy controls quantified by iTRAQ-2DLC-MS/MS.**

| **Protein ID** | **Abbreviation** | **Name** | **No. of peptides (>95%)** | **iTRAQ ratio (TB / Controls)** |
| --- | --- | --- | --- | --- |
| **Increased proteins** | |  |  |  |
| P00738 | HPT | Haptoglobin | 254 | 62.44 |
| P68871 | HBB | Hemoglobin subunit beta | 50 | 57.22 |
| P04003 | C4BPA | C4b-binding protein alpha chain | 32 | 20.99 |
| P0CG05 | LAC2 | Ig lambda-2 chain C regions | 10 | 20.44 |
| P01871 | IGHM | Ig mu chain C region | 17 | 16.44 |
| P00739 | HPTR | Haptoglobin-related protein | 95 | 14.72 |
| P01860 | IGHG3 | Ig gamma-3 chain C region | 19 | 14.67 |
| P69905 | HBA | Hemoglobin subunit alpha | 14 | 13.59 |
| P02741 | CRP | C-reactive protein | 9 | 12.64 |
| P01876 | IGHA1 | Ig alpha-1 chain C region | 10 | 11.77 |
| P02735 | SAA | Serum amyloid A protein | 30 | 9.34 |
| P20851 | C4BPB | C4b-binding protein beta chain | 17 | 8.81 |
| P02675 | FIBB | Fibrinogen beta chain | 12 | 8.39 |
| P01880 | IGHD | Ig delta chain C region | 8 | 8.30 |
| P02763 | A1AG1 | Alpha-1-acid glycoprotein 1 | 50 | 7.76 |
| P19652 | A1AG2 | Alpha-1-acid glycoprotein 2 | 26 | 6.96 |
| P01834 | IGKC | Ig kappa chain C region | 10 | 6.59 |
| P02042 | HBD | Hemoglobin subunit delta | 36 | 5.86 |
| P20742 | PZP | Pregnancy zone protein | 77 | 5.73 |
| Q15485 | FCN2 | Ficolin-2 | 12 | 4.54 |
| P10909 | CLUS | Clusterin | 39 | 3.80 |
| P07225 | PROS | Vitamin K-dependent protein S | 36 | 3.73 |
| P02766 | TTHY | Transthyretin | 5 | 3.58 |
| Q14520 | HABP2 | Hyaluronan-binding protein 2 | 10 | 3.54 |
| Q9BXR6 | FHR5 | Complement factor H-related protein 5 | 16 | 3.42 |
| O43866 | CD5L | CD5 antigen-like | 14 | 3.27 |
| P04278 | SHBG | Sex hormone-binding globulin | 32 | 2.90 |
| Q06033 | ITIH3 | Inter-alpha-trypsin inhibitor heavy chain H3 | 101 | 2.85 |
| P08571 | CD14 | Monocyte differentiation antigen CD14 | 21 | 2.62 |
| B9A064 | IGLL5 | Immunoglobulin lambda-like polypeptide 5 | 7 | 2.49 |
| Q15848 | ADIPO | Adiponectin | 11 | 2.39 |
| Q9UGM5 | FETUB | Fetuin-B | 20 | 2.30 |
| P11226 | MBL2 | Mannose-binding protein C | 18 | 2.25 |
| P37802 | TAGL2 | Transgelin-2 | 11 | 2.01 |
| P01031 | CO5 | Complement C5 | 198 | 1.97 |
| P24821 | TENA | Tenascin | 12 | 1.95 |
| Q15942 | ZYX | Zyxin | 3 | 1.86 |
| P02751 | FINC | Fibronectin | 64 | 1.85 |
| P18065 | IBP2 | Insulin-like growth factor-binding protein 2 | 5 | 1.80 |
| P18428 | LBP | Lipopolysaccharide-binding protein | 20 | 1.74 |
| P00918 | CAH2 | Carbonic anhydrase 2 | 4 | 1.68 |
| Q08380 | LG3BP | Galectin-3-binding protein | 9 | 1.64 |
| P01033 | TIMP1 | Metalloproteinase inhibitor 1 | 1 | 1.64 |
| P01344 | IGF2 | Insulin-like growth factor II | 3 | 1.63 |
| O00151 | PDLI1 | PDZ and LIM domain protein 1 | 7 | 1.63 |
| P10124 | SRGN | Serglycin | 11 | 1.62 |
| P07737 | PROF1 | Profilin-1 | 5 | 1.62 |
| Q9Y4L1 | HYOU1 | Hypoxia up-regulated protein 1 | 5 | 1.54 |
| Q9BWP8 | COL11 | Collectin-11 | 5 | 1.48 |
| P10643 | CO7 | Complement component C7 | 113 | 1.35 |
| P22891 | PROZ | Vitamin K-dependent protein Z | 2 | 1.31 |
| **Decreased proteins** | |  |  |  |
| P17936 | IBP3 | Insulin-like growth factor-binding protein 3 | 10 | 0.76 |
| P40197 | GPV | Platelet glycoprotein V | 20 | 0.73 |
| P07477 | TRY1 | Trypsin-1 | 25 | 0.73 |
| O00391 | QSOX1 | Sulfhydryl oxidase 1 | 25 | 0.72 |
| Q14515 | SPRL1 | SPARC-like protein 1 | 6 | 0.71 |
| P06276 | CHLE | Cholinesterase | 21 | 0.70 |
| P00746 | CFAD | Complement factor D | 12 | 0.66 |
| P13591 | NCAM1 | Neural cell adhesion molecule 1 | 10 | 0.66 |
| P00748 | FA12 | Coagulation factor XII | 36 | 0.64 |
| P24593 | IBP5 | Insulin-like growth factor-binding protein 5 | 1 | 0.62 |
| Q96S96 | PEBP4 | Phosphatidylethanolamine-binding protein 4 | 2 | 0.62 |
| P02787 | TRFE | Serotransferrin | 216 | 0.61 |
| P03952 | KLKB1 | Plasma kallikrein | 54 | 0.60 |
| P43251 | BTD | Biotinidase | 23 | 0.58 |
| P02654 | APOC1 | Apolipoprotein C-I | 19 | 0.57 |
| P05452 | TETN | Tetranectin | 40 | 0.56 |
| P13671 | CO6 | Complement component C6 | 83 | 0.54 |
| P36955 | PEDF | Pigment epithelium-derived factor | 157 | 0.50 |
| O95445 | APOM | Apolipoprotein M | 9 | 0.50 |
| P02753 | RET4 | Retinol-binding protein 4 | 79 | 0.48 |
| P43652 | AFAM | Afamin | 65 | 0.47 |
| P55056 | APOC4 | Apolipoprotein C-IV | 7 | 0.45 |
| P22105 | TENX | Tenascin-X | 25 | 0.43 |
| P08833 | IBP1 | Insulin-like growth factor-binding protein 1 | 1 | 0.43 |
| P02775 | CXCL7 | Platelet basic protein | 26 | 0.43 |
| P02788 | TRFL | Lactotransferrin | 10 | 0.20 |
| P02655 | APOC2 | Apolipoprotein C-II | 47 | 0.18 |
| P02768 | ALBU | Serum albumin | 27 | 0.02 |

We quantified 79 abnormally expressed proteins between patients with TB and healthy controls including 51 up-regulated proteins (fold changes >1.25) and 28 down-regulated proteins (fold changes <0.8).
