# Supplementary figures and images for: Serum amyloid A, protein Z, and C4b-binding protein β chain as new potential biomarkers for pulmonary tuberculosis
Source: PLoS One. 2017 Mar 9;12(3):e0173304. doi: 10.1371/journal.pone.0173304 (PMC5344400; doi:10.1371/journal.pone.0173304)

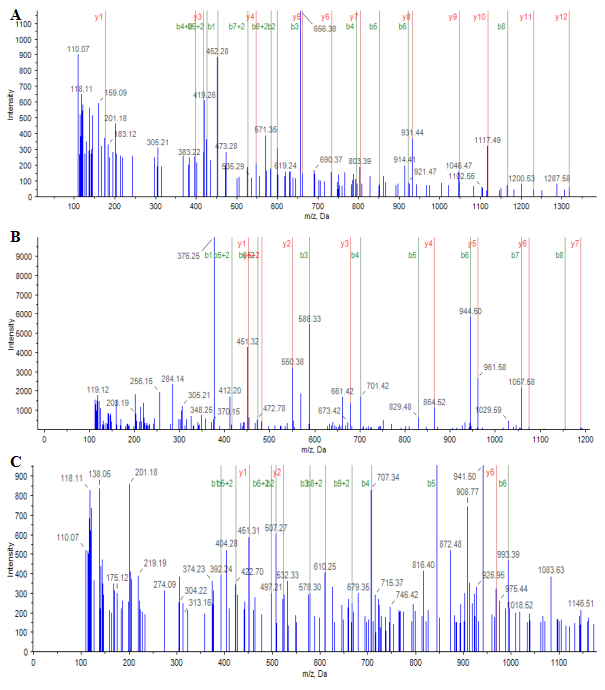

Supplement: S1 Fig — (A) The peptide sequence FFGHGAEDSLADQAANEWGR for SAA identification. (B) The sequence APDLQDLPWQVK for the identification of PROZ. (C) The peptide sequence SDAEHCPELPPVDNSIFVAK for C4BPB identification. The ion assignments were as follows: 113, healthy controls; 115, pneumonia group; 117, COPD group; 119, TB group. Note: 114, 116, 118, and 121 were used for other diseases not included in this study. (TIF) [file pone.0173304.s003.tif]
